# Neuroinvasive *Onchocerca lupi* Infection in a Ten-Year-Old Girl

**DOI:** 10.1155/2022/9773058

**Published:** 2022-12-05

**Authors:** Dorothy Bowers Wu, Brandon Ko, Gloria Lopez Hernandez, James Botros, Heather Spader, Sarah Sapp, Yvonne Qvarnstrom, Christopher D. Paddock, Paul T. Cantey, Walter Dehority

**Affiliations:** ^1^The University of New Mexico School of Medicine, Albuquerque, NM, USA; ^2^The University of New Mexico School of Medicine, Department of Pediatrics, Albuquerque, NM, USA; ^3^The University of New Mexico School of Medicine, Department of Pediatrics, Division of Critical Care, Albuquerque, NM, USA; ^4^The University of New Mexico School of Medicine, Department of Neurosurgery, Albuquerque, NM, USA; ^5^The Centers for Disease Control and Prevention, Division of Parasitic Diseases and Malaria, Atlanta, GA, USA; ^6^The Centers for Disease Control and Prevention, Division of High-Consequence Pathogens and Pathology, Atlanta, GA, USA; ^7^The University of New Mexico School of Medicine, Department of Pediatrics, Division of Infectious Diseases, Albuquerque, NM, USA

## Abstract

The nematode *Onchocerca lupi* is an emerging human pathogen. Though its life cycle is not well studied, it likely infects humans after a bite from a black fly vector, which in turn acquires infective microfilariae from an infected canid. These microfilariae mature into an infective larval stage within the fly. Among six reported cases in the United States, five involved children, and all occurred in the southwest. In this report, we present a case of *O. lupi* infection with cervical spine invasion in a healthy 10-year-old girl. She presented with five months of neurological symptoms from a rural and medically underserved area, highlighting a need for clinical vigilance in such settings for this emerging infectious threat in the American southwest.

## 1. Introduction

*Onchocerca lupi*, a nematode related to *Onchocerca volvulus* and infecting canids (and possibly felids) as the definitive host, may rarely infect humans [1–3]. Though some aspects of its life cycle remain unknown, the parasite is believed to infect humans following the bite of an infective insect black fly vector. The vector acquires parasite microfilariae from the infected canid, which mature into the infective larval stage within the insect. Infections in black flies, domestic dogs, wild coyotes, and humans have been identified in the United States over the last decade. [1, 3, 4]. Among the six cases in the United States reported in the literature, five involved children, and all occurred in southwestern states. Pathogenesis of disease with *O. lupi* frequently involves deep-seated lesions, such as those in the cervical spine [1, 5, 6]. Neuroinvasion such as this is responsible for a majority of severe symptoms present in *O. lupi* infection [1]. This may lead to a variety of symptoms at presentation, which may mimic more common diagnoses, such as meningitis or pharyngitis [1, 5, 6]. Without neuroinvasion, the presentation is often mild and insidious.

Herein, we present a case of *O. lupi* infection invading the cervical spine in a previously healthy 10-year-old girl.

## 2. Case Report

A 10-year-old Native American girl from northern New Mexico presented with a 5-month history of intermittent neck, right arm, and shoulder pain with progressive right arm weakness. Approximately one month prior to admission, she developed left leg weakness causing multiple falls weekly, progressive visual changes, and aphasia. The girl was afebrile and well appearing. She demonstrated weakness in her right deltoid, bicep, and hand. Right-sided pronator drift, Hoffmann's reflex and Babinski's reflex, and narrow-based gait were noted. No skin nodules were seen. Her peripheral white blood cell count was 14.8 cells × 103/*μ*L, with 88% polymorphonuclear cells, 9% lymphocytes, and 3% monocytes.

**Figure 1:**
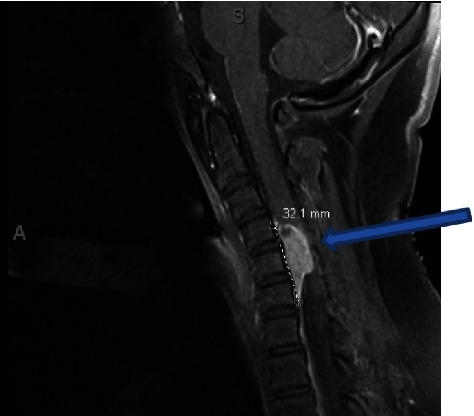
Sagittal T1-weighted preoperative MRI with contrast showing an anterior intradural extramedullary mass, measuring 1.6 × 0.9 × 2.8 cm (blue arrow). The mass is centered at C5 and spans from C4-6 with what appears to be a dural tail. The mass shows significant compression of the spinal cord.

Contrasted magnetic resonance imaging (MRI) of the brain was unremarkable. A contrasted MRI scan of the cervical spine revealed a 2.8 cm anterior intradural, extramedullary enhancing mass without restricted diffusion. A dural tail, centered at the level of the fifth cervical vertebra, was present, with enhancement and cord compression (Figure 1). Angiography demonstrated patent major cervical arteries. Testing for tuberculosis, *Coccidioides immitis*, *Bartonella henselae*, *Histoplasma* spp., and *Cryptococcus* spp. was negative. A chest X-ray scan was unremarkable. C-reactive protein, erythrocyte sedimentation rate, and lumbar puncture were not obtained at admission.

The patient underwent resection of the mass, with resultant anterior C5 and C6 corpectomies, C4-C5 and C6-C7 discectomies, and C4-C7 arthrodesis. The lesion was extremely adherent to the dura, requiring dissection and mobilization from the nerve root and spinal cord. Histopathology demonstrated fibrous tissue with caseating granulomas, with negative Fite's, Grocott–Gomori methenamine silver (GMS), and Gram stains. On postoperative day nine, a large pseudomeningocele developed in the neck, which improved following insertion of a lumbar drain.

**Figure 2:**
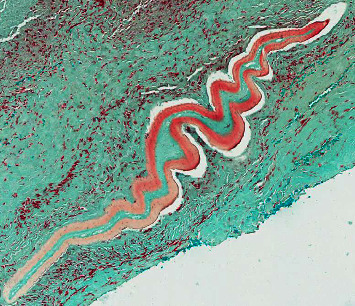
Longitudinal section revealing degenerating *Onchocerca lupi* nematode encased in inflamed, dense fibrous tissue. Lillie–Twort Gram stain, original magnification ×25.

**Figure 3:**
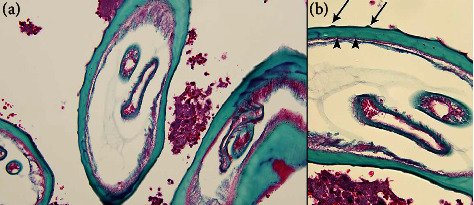
Trichrome-stained sections of *O. lupi* revealing relevant morphological features ((a) 200x magnification and (b) 400x magnification). Arrows indicate external cuticular ridges, and arrowheads indicate internal striae.

The patient resided in an area likely endemic for *O. lupi* [1]. Black flies of the *Simulium* genus reside in the state along the Rio Grande river, with increased numbers noted during warm weather months [7]. However, our patient denied exposure to putative risk factors for this infection (no exposure to bodies of freshwater, to areas where there are dogs with ocular disease, or to black fly bites). Initially, no helminth was seen, but a tissue specimen was sent to the Centers for Disease Control and Prevention. Fragments of inflamed, dense fibrous connective tissue contained abundant granulomas with irregular borders and areas of central necrosis. Intervening stroma contained strands of inflammatory infiltrates comprising plasma cells and macrophages, admixed with hemorrhage and occasional eosinophils. Within a few fragments of tissue were remnants of foreign material compatible with the cuticular wall of a degenerating nematode (Figure 2), for which morphological details could be emphasized using a trichrome stain. The outer cuticular ridges were dome-shaped and consistent with filariae in the genus *Onchocerca*. Other features typical of *Onchocerca* spp. that were evident included a thick hypodermis, weakly developed muscle cells rather vacuolated in appearance, and broad lateral chords. Longitudinal sections of the cuticular wall revealed two inner striae per ridge, morphologically compatible with *O. lupi*, likely damaged by host immune response (Figure 3). No microfilariae were identified. The polymerase chain reaction test of these anatomic specimens definitively confirmed the species' identity as O. *lupi*.

The patient was treated with a single dose of ivermectin (12 mg), followed one week later by a six-week course of doxycycline (200 mg once daily). After completing a 26-day hospital stay (with 21 days postoperatively in the pediatric intensive care unit), she was discharged with slightly decreased strength in her right arm. She returned to a normal neurological examination at a two-month follow-up visit in the infectious disease clinic. A contrasted MRI scan and plain films of the cervical spine five months after discharge revealed only postsurgical changes.

## 3. Discussion

Our patient is the seventh documented case of *O. lupi* in the United States and the fourth involving the spine (Table 1). As with the previous spinal cases, the cervical spine of a child was involved [1]. Overlying cutaneous findings were absent, and fevers were not reliably present. The previous three cases presented after four weeks to several months of symptoms [1]. Our patient presented five months after symptom onset, which is the longest period of time reported for a child with *O. lupi* in the United States. Children with *O. lupi* may also be at risk for a delayed presentation for care and diagnosis due to their residence in rural, underserved regions of the southwestern United States, where the parasite is likely endemic. Our patient lived in a rural, medically underserved area four hours from the nearest tertiary medical center. Pediatric providers (and their virtual consultants) should have a high index of suspicion for *O. lupi* in the setting of new onset, persistent neurological abnormalities in an otherwise well-appearing child residing in the southwestern regions of the United States. Ascertainment of risk factors may be of assistance in raising the level of suspicion, and inquiries into a history of black fly bites, activities near bodies of freshwater and streams likely to harbor insect vectors, and cohabitation with dogs with ocular disease may be helpful, but all may be absent as they were for our patient [1].

Most reported human *O. lupi* infections outside the United States appear to consist almost entirely of subconjunctival and localized ocular disease in adults, while infection in the United States predominately affects children, with a high risk of neuroinvasion [1]. Of the seven reported cases of human *O. lupi* infection in the United States, six involved children, and four of those had involvement of the cervical spine [1]. No other onchocercal parasite, including *O. volvulus*, is known to have a similar tendency to affect the central nervous system, though *O. lupi* is typically found in the head in canids [3]. Three children presented with intradural and one with extradural involvement, suggesting that *O. lupi* may traverse the dura once within the spinal canal. Despite lacking typical lymphatic drainage, recent research demonstrates the existence of lymphatic vessels lining dural sinuses, with connections to cervical lymph nodes [8]. This may represent a mechanism by which *O. lupi* traverses the skin following a cutaneous insect bite on exposed regions of the neck and enters the central nervous system. Such lymphatics do not appear to infiltrate brain parenchyma, which may further explain the current absence of intraparenchymal brain lesions in such infections [9]. Significant declines in central nervous system lymphatic drainage are observed in aged mice [9], which could explain why children appear more susceptible to central nervous system invasion by *O. lupi* than adults.

Suspicion of *O. lupi* nodules in any anatomical location may be evaluated with MRI, computed tomography, or ultrasound. However, differentiation of *O. lupi* infection from tumors or other invasive processes is difficult on radiological grounds alone. Excision of masses and skin biopsies are needed for debulking of infection and identification of the organism, given the current absence of an available serologic test. Morphologic examination of the excised material is important for identification of the causative agent, particularly in distinguishing *O. lupi* from other zoonotic *Onchocerca* spp., such as *O. cervicalis*, or any other helminthic agents, which may present with a similar clinical picture. Limited reports in humans and dogs suggest that incomplete excision may be associated with relapse of symptoms [1]. Treatment is currently based on surgical excision for parasite identification via histopathology and the polymerase chain reaction test, followed by medical therapy that is based on standard approaches for the management of *O. volvulus* infections, with ivermectin as an antihelminthic agent to kill microfilariae and doxycycline to target endosymbiont *Wolbachia spp.* required for survival of adult forms of the parasite [1]. Corticosteroids may be considered for control of inflammation in central nervous system infection following anti-infective treatment, but such practice is currently based on theoretical and anecdotal evidence. An ophthalmological slit-lamp examination to evaluate for microfilariae prior to initiating ivermectin treatment is unlikely to be helpful in low intensity infections, but an exam to identify subconjunctival nodules may help establish the diagnosis.

Additional research is needed to better define the burden of the disease in the United States, including evaluation of potential vector species and their habitats, starting where human or canid infections have been identified, and perhaps additional case finding among domestic dog populations. Development of serological assays would allow for assessment of seroprevalence in endemic regions in humans and dogs and help quantify the burden of asymptomatic infection. Understanding the distribution of the infection, risk factors for exposure, and risk for severe infections are key toward developing targeted prevention strategies.

## Figures and Tables

**Table 1:** Summary of reported cases of human *Onchocerca lupi* infection in the United States.

	Case						
	1	2	3	4	5	6	7^*∗*^
Gender	Female	Female	Female	Male	Female	Male	Female
Age (years)	1.8	10	50	13	5	10	10
Nodule location	C-spine	Scalp	Forearm	C-spine	C-spine rectus muscle	Superior	C-spine
Gravid adult?	Yes	No	No	No	No	Yes	No
Treatment	Ivermectin	Excision	E/I/D	E/I/D	E/I/D	E/I/D	E/I/D
Outcome	Good	Good	Good	Good	Good	Good	Good

^*∗*^Current case. C-spine, cervical spine; E/I/D = excision, ivermectin, and doxycycline. Table adapted from reference [1].

## Data Availability

All relevant data are included in the article.
